# Increased Atherosclerosis in HIV-Infected Humanized Mice Is Caused by a Single Viral Protein, Nef

**DOI:** 10.1093/infdis/jiaf192

**Published:** 2025-04-16

**Authors:** Yongsen Wang, Beda Brichacek, Larisa Dubrovsky, Tatiana Pushkarsky, Kyle Korolowicz, Olga Rodriguez, Yichien Lee, Marta Catalfamo, Chris Albanese, Anastas Popratiloff, Dmitri Sviridov, Michael Bukrinsky

**Affiliations:** Department of Microbiology, Immunology and Tropical Medicine, The George Washington University School of Medicine and Health Sciences, Washington, District of Columbia, USA; Department of Microbiology, Immunology and Tropical Medicine, The George Washington University School of Medicine and Health Sciences, Washington, District of Columbia, USA; Department of Microbiology, Immunology and Tropical Medicine, The George Washington University School of Medicine and Health Sciences, Washington, District of Columbia, USA; Department of Microbiology, Immunology and Tropical Medicine, The George Washington University School of Medicine and Health Sciences, Washington, District of Columbia, USA; Department of Oncology, Center for Translational Research, Georgetown University Medical Center, Washington, District of Columbia, USA; Department of Radiology, Lombardi Comprehensive Cancer Center and Center for Translational Research, Georgetown University Medical Center, Washington, District of Columbia, USA; Department of Oncology, Center for Translational Research, Georgetown University Medical Center, Washington, District of Columbia, USA; Department of Radiology, Lombardi Comprehensive Cancer Center and Center for Translational Research, Georgetown University Medical Center, Washington, District of Columbia, USA; Department of Oncology, Center for Translational Research, Georgetown University Medical Center, Washington, District of Columbia, USA; Department of Radiology, Lombardi Comprehensive Cancer Center and Center for Translational Research, Georgetown University Medical Center, Washington, District of Columbia, USA; Department of Microbiology and Immunology, Georgetown University School of Medicine, Washington, District of Columbia, USA; Department of Oncology, Center for Translational Research, Georgetown University Medical Center, Washington, District of Columbia, USA; Department of Radiology, Lombardi Comprehensive Cancer Center and Center for Translational Research, Georgetown University Medical Center, Washington, District of Columbia, USA; George Washington University Nanofabrication and Imaging Center, The George Washington University, Washington, District of Columbia, USA; Laboratory of Lipoproteins and Atherosclerosis, Baker Heart and Diabetes Institute, Melbourne, Victoria, Australia; Department of Biochemistry and Molecular Biology, Monash University, Clayton, Victoria, Australia; Department of Microbiology, Immunology and Tropical Medicine, The George Washington University School of Medicine and Health Sciences, Washington, District of Columbia, USA

**Keywords:** HIV, Nef, atherosclerosis, humanized mice, inflammation

## Abstract

Antiretroviral therapy suppresses human immunodeficiency virus (HIV) replication, reverses immunodeficiency, and reduces AIDS-related symptoms, but non-AIDS comorbidities like cardiovascular diseases remain a major challenge for people with HIV (PWH). The pathogenic mechanisms driving these comorbidities are poorly understood. We previously showed that the HIV protein Nef contributes to chronic inflammation in PWH. Here, we explored Nef's role in HIV-associated atherosclerosis using a novel model: HIV-infected humanized mice expressing a gain-of-function mutant of proprotein convertase subtilisin/kexin type 9 (PCSK9) and fed a high-fat diet. Comparing atherosclerosis in uninfected mice to those infected with Nef-positive or Nef-deficient HIV-1, we found that Nef exacerbates atherosclerotic changes by increasing inflammation. These results identify Nef as a key driver of HIV-related atherosclerosis and provide a platform for testing therapeutic interventions targeting Nef to mitigate cardiovascular risks in PWH.

## Teaser

This article presents a mouse model of HIV-induced atherosclerosis and identifies HIV Nef as the key driver of HIV-associated atherogenesis. It demonstrates that Nef's primary atherogenic mechanism is the stimulation of inflammation.

The introduction of effective antiretroviral therapy (ART) has drastically improved human immunodeficiency virus (HIV) outcomes, allowing people with HIV (PWH) to achieve life expectancies comparable to people without HIV. Clinical concerns have shifted from managing immunodeficiency to mitigating non-AIDS comorbidities, particularly cardiovascular issues like atherosclerosis, which grow as the HIV population ages [1, 2]. Atherosclerosis is influenced by genetic, environmental, dietary, and infectious factors [3]. In the context of HIV infection, the viral protein Nef contributes to atherosclerosis by inducing inflammation, suppressing cholesterol efflux, and inhibiting autophagy [4–7]. Evidence from ApoE^−/−^ mice injected with recombinant Nef and Nef-transgenic mice demonstrates Nef's proatherogenic effects [5, 8]. However, direct evidence linking Nef to atherosclerosis in HIV infection is limited due to challenges combining atherosclerosis-susceptible phenotypes with HIV susceptibility in mice. To address this, we applied an approach using PCSK9 gain-of-function mutant to induce dyslipidemia in mice fed a high-fat diet. This strategy effectively promotes the development of atherosclerosis in C57BL/6 mice [9].

In this study, we utilized this approach in immunodeficient mice humanized with human CD34^+^ hematopoietic progenitor cells and infected with HIV. Our findings reveal that these mice develop systemic inflammation and aortic changes consistent with enhanced atherogenesis. Notably, Nef emerged as the key driver of enhanced atherogenesis in HIV-infected mice.

## METHODS

### Mouse Model

Humanized NSG mice (NOD.Cg-Prkdcscid IL2rgtm1Wjl/SzJ; Jackson Laboratory) were engrafted with human cord blood CD34^+^ hematopoietic stem/progenitor cells (Lonza). Engraftment was confirmed by flow cytometry at 10 weeks, with mice showing >20% human CD4^+^ cells used for HIV infection. Mice received AAV8/D377Y-mPCSK9 (Vector Biolabs) via tail vein injection (5.0 × 10^11^ viral genomes/mouse) and were fed a western-type diet (Research Diets) for 13 weeks. Controls included humanized mice on the diet without AAV-PCSK9. Studies were approved by George Washington University Institutional Animal Care and Use Committee (A2023-057).

### HIV-1 Strain and Infection

Mice were infected intraperitoneally with wild-type (HIVwt) or Nef-deficient (HIVΔNef) HIV-1 JR-CSF variants [10] (8.0 × 10^5^ or 6.8 × 10^5^ pg p24/mouse, respectively). The JR-CSF strain of HIV-1 is a CCR5-tropic clade B isolate from the cerebrospinal fluid (CSF) of an individual with HIV [11].

### Western Blot Analysis

HEK293T cells transfected with HIVwt or HIVΔNef were lysed and analyzed using capillary western blotting (Jess Bio-Techne) with anti-Nef antibodies (Abcam) as described [12].

### Flow Cytometry

Whole blood was collected, red blood cells lysed, and white blood cells stained with anti-human CD45, CD3, CD4, CD8, and CD14 antibodies (Biolegend, BD Pharmingen). Analysis was performed on a BD Celesta using FlowJo (Supplementary Figure 1).

### Analysis of HIV Load in Plasma

Plasma viremia was assessed biweekly by quantitative polymerase chain reaction (qPCR; SYBR Green; Bio-Rad) using JR-CSF–specific primers. Murine β-globin was used as a loading control.

### PCSK9 and Cytokines

Plasma PCSK9 was measured by enzyme-linked immunosorbent assay (ELISA; R&D Systems). Cytokines (murine tumor necrosis factor-α [TNF-α], murine interleukin 6 [IL-6], murine IL-1β, and human TNF-α) were analyzed using R&D Systems ELISA kits.

### Lipoproteins

Total cholesterol, low-density lipoprotein cholesterol (LDL-C), high-density lipoprotein cholesterol (HDL-C), and triglycerides were measured using Fujifilm/WAKO and Crystal Chem kits.

### Aorta Analysis

Aortas were stained with Oil Red O (ORO) and imaged. Plaque areas were quantified using ImagePro Premier. Spectral confocal imaging was performed with Zeiss 980 confocal microscope equipped with 32-channel spectral detector and Coherent Discovery Optical Parametric Oscillator, producing 80 MG Hz output with pulse duration of 100 fsec. Ultrasound analysis was done using the VisualSonics VEVO 3100 instrument and VisualSonics analysis software Vevo LAB version 5.8.2. Magnetic resonance imaging (MRI) was performed on a Bruker 7T/30 AVANCE NEO/ParaVision 360 scanner (S10OD025153).

### Statistical Analysis

All values are presented as mean ± SD. Group comparisons were performed using 1-way ANOVA with Tukey correction for multiple comparisons when data followed a normal distribution, as assessed by the Shapiro-Wilk normality test. For data that did not meet the assumption of normality, the Kruskal-Wallis test with Dunn post hoc correction for multiple comparisons was used. Correlation analysis was conducted using GraphPad Prism (version 10). A *P* value of <.05 was considered statistically significant.

## RESULTS

### Mouse Model of HIV-Associated Atherosclerosis

**Figure 1.**
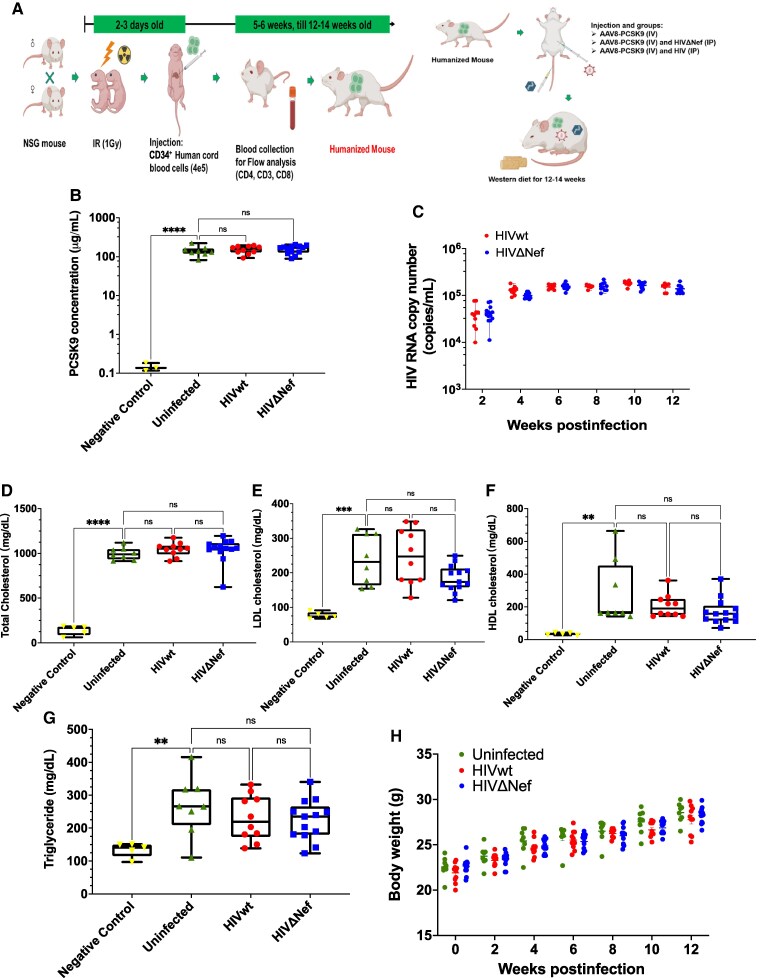
Mouse model of HIV-associated atherosclerosis. *A*, Experimental procedure schematic. *B*, PCSK9 concentrations at euthanasia. Results shown as box plots (mean ± SD). Groups compared using 1-way ANOVA with Tukey test. *C*, HIV RNA quantified every 2 weeks by qRT-PCR. *D*, Total cholesterol measured using Wako kit. Results analyzed as in (*B*). Negative control was humanized mice fed a high-fat diet without AAV-PCSK9. *E*, LDL-C levels measured and analyzed as in (*B*). *F*, HDL-C levels measured using Crystal Chem kit. Results are presented as in (*B*). Results analyzed using Kruskal-Wallis test with Dunn correction. *G*, Triglycerides measured using Wako kit. Results analyzed as in (*B*). *H*, Body weight measured every 2 weeks. Box plots show the median (line), interquartile range (box), and minimum/maximum values (whiskers). ****P < .0001, ***P < .001, **P < .01, *P < .05. Abbreviations: HDL-C, high-density lipoprotein cholesterol; HIVwt, HIV wild type; HIVΔNef, Nef-deficient HIV; IP, intraperitoneal; IR, irradiation; IV, intravenous; LDL-C, low-density lipoprotein cholesterol; ns, not significant; qRT-PCR, quantitative reverse transcription polymerase chain reaction.

Immunodeficient NSG mice were humanized by injecting human CD34^+^ cord blood cells into newborn γ-irradiated pups [13]. To induce atherosclerosis susceptibility, mice received AAV8 expressing a gain-of-function mutant of murine PCSK9 (AAV-PCSK9) and were fed a high-fat western diet for 12–14 weeks (Figure 1*A*). A negative control group consisted of humanized mice fed a western diet but not injected with AAV-PCSK9. AAV-PCSK9–injected mice exhibited much higher PCSK9 levels than controls (Figure 1*B*). One week postinjection, mice were infected with Nef-positive (HIVwt) or Nef-deficient (HIVΔNef) HIV-1 JR-CSF or left uninfected. Viral loads were comparable across infected groups (Figure 1*C*). All AAV-PCSK9–injected mice had elevated cholesterol and LDL-C levels, while HDL-C and triglycerides showed less pronounced differences (Figure 1*D*–*G*). No significant weight differences were observed (Figure 1*H*). Cumulative data are given in Table 1.

**Table 1.** Characteristics of Mice Used in the Study

Mouse ID^a^	Group	Sex	PCSK9, mg/dL^b^	hCD45, %^c^	hCD4, %^c^	hCD14, %^c^	logVL^b^
**530**	Uninfected	M	16.7	45.9	78.6	4.7	Neg
**533**	Uninfected	M	22.7	53.4	51.2	1.8	Neg
**532**	Uninfected	M	8.2	8.48	38.5	4.5	Neg
**546**	Uninfected	F	13.1	5.38	71.7	1.3	Neg
**534**	Uninfected	M	11.8	16.7	50.5	1.3	Neg
549	Uninfected	F	15.1	33.2	60.5	0.7	Neg
548	Uninfected	F	12.8	6.26	41.9	0.8	Neg
547	Uninfected	F	13.8	16.7	41.7	8.1	Neg
**571**	HIVwt	M	18.5	21.4	53.8	3.2	5.2
**572**	HIVwt	M	17.6	23.9	51.6	1.9	5.0
**573**	HIVwt	M	17.1	11.9	38.9	0.5	5.3
**553**	HIVwt	M	13.4	11.9	61.4	0.4	5.0
**574**	HIVwt	F	13.2	19.9	61.4	0.7	5.2
552	HIVwt	F	19.6	47.4	N/M^d^	2.9	5.2
554	HIVwt	F	18.6	33.2	51.9	3.1	5.2
550	HIVwt	F	11.6	60.7	52.3	1.7	5.2
510	HIVwt	M	9.3	47.0	97.6	0.1	5.2
511	HIVwt	F	14.6	15.5	71.3	1.9	5.2
**509**	HIVΔNef	M	18.5	56.6	56.4	1.5	5.2
**521**	HIVΔNef	F	17.6	10.5	77.5	0.7	5.0
**528**	HIVΔNef	F	17.1	41.0	57.1	1.1	5.3
**547**	HIVΔNef	F	13.4	22.5	73.6	0.1	5.0
**549**	HIVΔNef	F	13.2	4.2	44.3	4.4	5.0
551	HIVΔNef	M	20.7	8.4	16.7	1.7	5.1
527	HIVΔNef	F	17.3	52.9	57.9	1.4	5.1
525	HIVΔNef	F	12.1	13.0	71.9	1.8	5.1
526	HIVΔNef	F	18.5	12.0	50.0	1.1	5.1
581	HIVΔNef	M	8.9	21.6	NM	1.6	5.2
582	HIVΔNef	M	10.2	27.8	62.3	2.5	5.2
583	HIVΔNef	M	16.4	32.9	65.9	3.6	5.1
584	HIVΔNef	M	19.8	27.6	56.5	1.3	NM

Abbreviations: F, female; HIVwt, human immunodeficiency virus wild type; HIVΔNef, human immunodeficiency virus Nef-deficient; M, male; NM, not measured; VL, viral load.

^a^ID numbers of mice used for Oil Red O staining are shown in bold.

^b^PCSK9 levels and VL were measured at the time of euthanasia.

^c^The percentage of human cells was measured at week 10 postreconstitution of irradiated mice with human CD34^+^ cells.

### Development of Atherosclerosis

**Figure 2.**
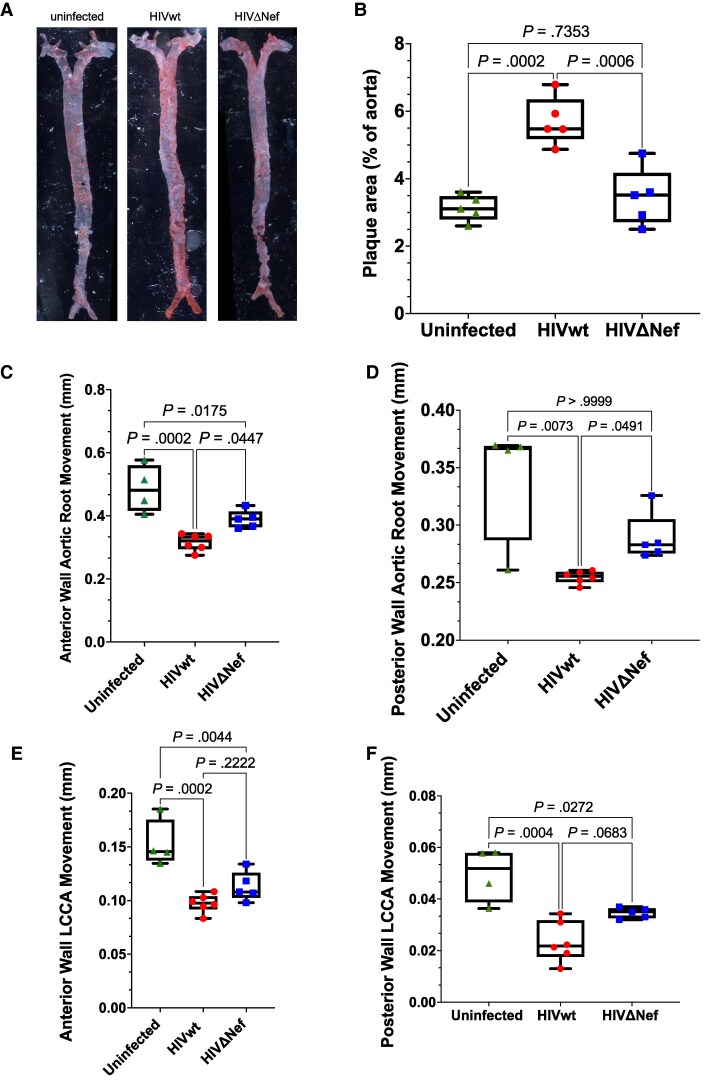
Vascular atherosclerosis analysis. *A*, En face ORO staining of aorta. *B*, Quantitative staining analysis. Results shown as box plots (mean ± SD). Groups compared using 1-way ANOVA with Tukey test. *C–F*, Vascular ultrasound analysis. Imaging performed in M Mode using a 40-MHz transducer. Wall displacement averaged over 3 cardiac cycles. Error bars indicate SD. *C*, Anterior aortic root displacement. Groups compared using 1-way ANOVA with Tukey test. *D*, Posterior aortic root displacement. Analysis by Kruskal-Wallis test with Dunn correction. *E*, Anterior LCCA displacement. Analysis as in (*C*). *F*, Posterior LCCA displacement. Analysis as in (*C*). n = 4 (uninfected), n = 6 (HIVwt), n = 5 (HIVΔNef). Abbreviations: HIVwt, HIV wild type; HIVΔNef, Nef-deficient HIV; LCCA, left common carotid artery. Box plots show the median (line), interquartile range (box), and minimum/maximum values (whiskers).

En face staining of the aorta with ORO revealed more lipid-rich regions in HIVwt-infected mice compared to controls and HIVΔNef-infected mice (Figure 2*A* and 2*B*), highlighting Nef's role in atherogenesis.

Ultra-high frequency ultrasound (UHFUS) analysis [14] of the aortic root and left common carotid artery showed decreased artery wall movement in HIVwt-infected mice relative to uninfected or HIVΔNef-infected mice, and in HIVΔNef-infected mice relative to uninfected (Figure 2*C*–*F*). These findings suggest Nef and non-Nef HIV-derived factors contribute to arterial stiffness [15]. Other cardiac function measures (eg, cardiac output, left ventricular wall thickness) did not significantly differ between groups (Supplementary Figure 2*A–D*).

Mice were also imaged using the CINE IG (inversion recovery gradient echo) FLASH (fast low angle shot) MRI technology, which allows assessment of dynamic processes, such as blood flow patterns, wall motion, and heart function [16]. Analysis of these datasets using a region of interest localized in the aortic arch at the base of the carotids (Supplementary Figure 3*A*) revealed an increased mean intensity of the region of interest in mice infected with Nef-positive, but not Nef-deficient, HIV-1 compared to the uninfected group (Supplementary Figure 3*B* and 3*C*). This result may be secondary to a narrowed and less elastic aorta due to atherosclerotic changes. This conclusion is consistent with a trend towards decreased cardiac output in HIVwt-infected group (Supplementary Figure 2*A*).

**Figure 3.**
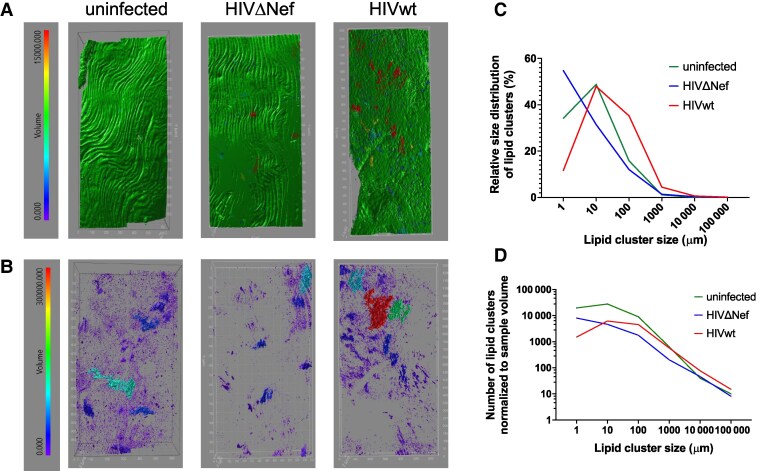
Two-photon imaging of aorta. Aortas imaged using 25×/0.8 oil immersion objective. *A*, ORO labeling surface model. *B*, ORO plus elastin autofluorescence. Lipid cluster size is color coded by volume (red, largest; blue, smallest). Image volume stitched from 2 × 3 (uninfected, HIVΔNef) or 4 × 3 (HIVwt) fields. *C*, ORO-stained lipid cluster size distribution. *D*, ORO-stained lipid clusters per media volume. Abbreviations: HIVwt, HIV wild type; HIVΔNef, Nef-deficient HIV; ORO, Oil Red O.

Finally, to characterize atherogenic changes, we used 2-photon excitation and second-harmonic generation (SHG) with spectral imaging and linear unmixing. A single-field view volumes were taken with a higher resolution objective lens (Supplementary Figure 4*A*). The final images were taken with online spectral unmixing using generated spectral curves from areas presenting only SHG, autofluorescence, or ORO (Supplementary Figure 4*B*). Spectral imaging overcomes the extensive autofluorescence in the tissue, which overlaps with ORO labeling under conventional fluorescence imaging. The 960-nm short-pulse excitation for 2-photon microscopy enabled distinct collagen detection via SHG, identifiable by a sharp spectral peak at half the excitation wavelength (Supplementary Figure 4*B*). Collagen expression patterns distinguished the tunica media (Supplementary Figure 5*A*) from the adventitia (Supplementary Figure 5*A*). Autofluorescence, originating from cellular elements and elastic fibers, was distinguishable under high-resolution imaging (Supplementary Figure 4*B*). Confocal images were analyzed via 3-dimensional intensity segmentation, identifying ORO clusters within the aortic wall (Figure 3*A*) while excluding adventitial adipocytes, distinguished by collagen bundles and reduced autofluorescence (Supplementary Figure 5*A*). Images revealed increased ORO staining in HIVwt-infected mice compared to uninfected or HIVΔNef-infected mice (Figure 3*B*). Lipid cluster analysis showed smaller clusters (1–10 μm) in uninfected and HIVΔNef-infected mice but larger clusters (>100 μm) in HIVwt-infected mice (Figure 3*C* and 3*D*). HIVwt-infected mice exhibited disrupted endothelial and elastin layers, with irregular patterns absent in uninfected and HIVΔNef-infected mice (Figure 3*A*). Zoomed-in images of plaque-affected areas in HIVwt-infected mice confirm structural disruption in collagen and endothelium/elastin layers (Supplementary Figure 5*B*).

### Biochemical Analysis

Given Nef and Nef extracellular vesicles’ (EVs’) ability to downregulate ABCA1 and inhibit cholesterol efflux [17, 18], we hypothesized that HIVwt-infected mice would show reduced HDL-C levels [19]. Unexpectedly, no significant differences in HDL-C were found between uninfected, HIVwt-infected, and HIVΔNef-infected mice (Figure 1*F*). Liver ABCA1 expression was only insignificantly reduced in HIV-infected mice (Supplementary Figure 6*A*), nor did it correlate with viral load (Supplementary Figure 6*B*). As expected, LDL-C levels also showed no significant differences (Figure 1*C*). These results indicate that liver ABCA1 and HDL levels are not key contributors to the increased atherosclerosis in Nef-positive HIV infection.

**Figure 4.**
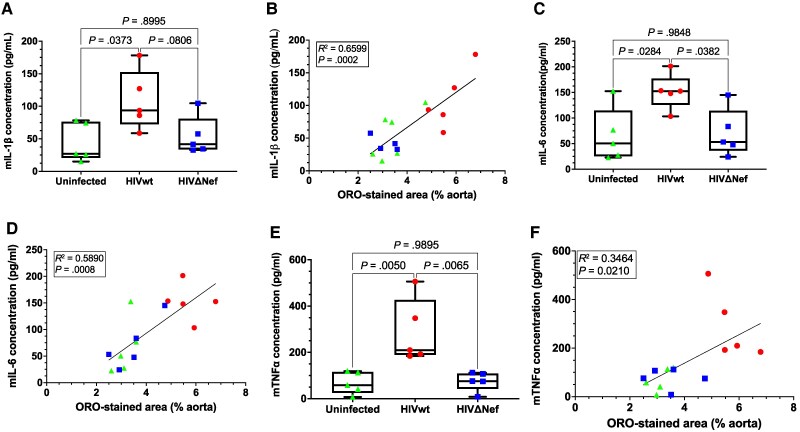
Cytokine levels in blood. *A*, Murine IL-1β levels. Results shown as box plots (mean ± SD). Groups compared using 1-way ANOVA with Tukey test. *B*, IL-1β versus ORO-stained area correlation. *C*, Murine IL-6 levels. *D*, IL-6 versus ORO-stained area correlation. *E*, Levels of murine TNF-α. Results are presented as in (*A*). *F*, TNF-α versus ORO-stained area correlation. All comparisons were made as in (*A*). Color and shape of the symbols in (*B*, *D*, and *F*) match (*A*, *C*, and *E*). Abbreviations: HIVwt, HIV wild type; HIVΔNef, Nef-deficient HIV; IL, interleukin; ORO, Oil Red O; TNF-α, tumor necrosis factor-α. Box plots show the median (line), interquartile range (box), and minimum/maximum values (whiskers).

Atherosclerosis is characterized by elevated inflammatory cytokines [20]. In mice, IL-1β (Figure 4*A*), IL-6 (Figure 4*C*), and TNF-α (Figure 4*E*) were significantly higher in those infected with HIVwt compared to uninfected and HIVΔNef groups, which showed no differences. Murine TNF-α, IL-1β, and IL-6 in the negative control group (NSG mice without AAV-PCSK9 injection) were below the ELISA detection threshold (1–5 pg/mL), consistent with NSG mice's severe immunodeficiency, which limits murine immune responses. Notably, human TNF-α levels were similar between Nef-positive and Nef-deficient HIV-infected groups (Supplementary Figure 7*A*), suggesting that Nef-mediated inflammation is primarily driven by murine factors. This is further supported by a strong correlation between ORO-stained areas and murine IL-1β (Figure 4*B*), IL-6 (Figure 4*D*), and TNF-α (Figure 4*F*), but not human TNF-α (Supplementary Figure 7*B*). To enhance reliability, the analyses in Figures 4*B*, 4*D*, and 4*F* were conducted using data points across all 3 groups, reducing variability associated with small sample sizes. Individual group data points are indicated in these figures.

These findings demonstrate that atherosclerosis progresses more severely in HIV-infected mice, with Nef as the key pathogenic factor driving inflammation in this model. This supports previous studies highlighting Nef 's proinflammatory effects on murine myeloid cells [19].

## DISCUSSION

This study shows that mice infected with HIVwt develop significantly more advanced atherosclerosis than those infected with HIVΔNef, whose changes resemble uninfected controls. Nef-driven effects were confirmed using ORO staining, 2-photon microscopy for intimal architecture, and UHFUS for real-time vascular imaging and arterial stiffness. Viral loads were comparable between HIVwt and HIVΔNef, ruling out replication differences as a cause. The lack of Nef's effect on replication likely reflects JR-CSF virus properties [10]. These findings establish Nef as the key HIV-associated driver of atherogenesis.

This study highlights key differences between the murine model of HIV-associated atherosclerosis used here and earlier models. Previous studies relied on artificial systems, such as transgenic mice expressing HIV or Nef proteins [8, 21], or injection of individual HIV proteins into genetically modified mice [5]. AAV-PCSK9 injection, which induces sustained PCSK9 expression, LDL receptor downregulation, and hyperlipidemia [22], combined with a high-fat diet, rapidly induces atherosclerosis in C57BL/6J mice [23]. However, prior efforts using AAV-PCSK9 in immunodeficient humanized NSG mice failed to generate significant atherosclerosis, likely due to insufficient cholesterol elevation (approximately 500 mg/dL [24] vs approximately 1500 mg/dL in the apoE^−/−^ model [25]). The genetic background also contributes, with C57BL/6J mice being more atherosclerosis prone than BALB/c, C3H, or NOD strains [26–29]. In this study, higher AAV-PCSK9 doses yielded cholesterol levels (approximately 1000 mg/dL) that addressed prior limitations, enhancing model reliability. Atherosclerotic plaques appeared approximately 13 weeks after a western diet but were smaller than those in C57BL/6J mice [22], representing early-stage plaque formation. Despite their smaller size, these lipid deposits disrupted aortic architecture and impaired function.

Atherosclerosis development in this study appears more dependent on murine than human cells, likely due to the low percentage (<5%) of human monocytes, which are critical for atherosclerosis in mice [30]. Because murine cells cannot be infected with HIV, the data suggest that Nef EVs released by HIV-infected human cells influence murine myeloid cells. Like human macrophages, murine cells are sensitive to Nef EVs, triggering robust inflammatory responses [5, 18, 31]. However, technical challenges in separating HIV virions from EVs [32, 33] prevented measuring plasma Nef EV levels.

Recombinant Nef injection accelerates atherosclerosis in ApoE^−/−^ mice by worsening dyslipidemia and inflammation [5]. In our study, however, no significant differences in lipid markers or suppression of hepatic ABCA1 were observed in HIVwt-infected mice, possibly due to PCSK9-mediated ABCA1 inhibition [34]. PCSK9 and Nef may interact through overlapping pathways, explaining the absence of HDL downregulation, a typical Nef-mediated outcome [5]. Thus, dyslipidemia likely does not drive HIV-associated atherosclerosis in this model. Instead, Nef EVs may locally inhibit cholesterol efflux and enhance vascular and systemic inflammation. Elevated IL-6, TNF-α, and IL-1β levels in HIVwt-infected mice support inflammation as the main mechanism behind Nef-dependent atherogenesis, consistent with the view of atherosclerosis as a chronic inflammatory disease [20].

Nef also activates cellular transcription factors that regulate HIV gene expression, thereby enhancing viral transcription [35]. In mice infected with HIVΔNef, this activation is reduced, potentially lowering the virus's proatherogenic impact. Additionally, Nef influences EV biogenesis, cargo selection, and the functional effects on recipient cells [36]. Given that EVs—whether or not they contain Nef—play a significant role in atherosclerosis pathogenesis [37, 38], this modulation likely contributes to the proatherogenic effects observed in Nef-positive HIV infection.

An interesting observation is the reduced number of smaller lipid clusters (<1 mm; Figure 3*B* and 3*C*) in mice infected with HIVΔNef relative to uninfected controls. This difference was not observed for larger clusters, which were similar between uninfected and HIVΔNef-infected mice but lower than in the HIVwt-infected group. This finding suggests a potential protective, antiatherogenic effect of mild immune activation in HIVΔNef-infected animals [39, 40], most evident during early plaque development.

The promotion of atherosclerosis by Nef aligns with its known atherogenic properties. In our model, the effects of other HIV proteins, such as gp120 and Tat, were not observed—atherosclerotic changes in HIVΔNef-infected mice were similar to uninfected controls. This suggests limited effects of these proteins on murine cells or poor incorporation of Tat into EVs [41]. In ART-suppressed HIV, circulating viral proteins rather than direct HIV infection influence comorbidities [42]. Nef appears to be the predominant viral protein circulating in ART-treated PWH, as only anti-Nef cytotoxic T lymphocyte responses are detected in long-term ART recipients [43]. In our model, with no direct infection of murine cells, EVs carrying viral proteins are the major pathogenic factor.

Mice, like all rodents, are naturally resistant to atherosclerosis, requiring both genetic modification and a dietary challenge to develop the disease. The humanized mice in this study were generated on the NOD (nonobese diabetic) background [44], a strain particularly resistant to atherosclerosis [29, 45]. Despite strong proatherogenic stimuli—PCSK9 overexpression and a high-fat diet—this model likely represents early-stage atherogenesis, comparable to that in middle-aged, clinically asymptomatic individuals, a common demographic among PWH. Although translating these findings to human disease is challenging due to inherent species differences and artificial modifications to enhance susceptibility, mouse models have historically provided critical insights into human atherosclerosis and led to successful therapeutic approaches [46, 47].

A limitation of this mouse model is the absence of human endothelial cells and the low abundance of human myeloid cells, which may have reduced the atherogenic activity of HIVΔNef. Enhancing mouse humanization techniques is needed to address this issue. This study focused on untreated HIV infection; however, because murine cells are resistant to HIV, the model effectively mimics ART-suppressed HIV in humans, where new infections are blocked. Unlike ART-treated PWH, HIV continues to circulate in this model, potentially amplifying the atherogenic effect. Future studies replicating true HIV latency will better reflect atherogenesis in ART-treated PWH. Another limitation of the study is the limited number of animals. Anticipating a medium-sized effect, we applied a heuristic rule to estimate sample size for comparing 3 groups: uninfected, HIVwt-infected, and HIVΔNef-infected. However, the observed effects were smaller than expected, leading to trends rather than definitive results in some experiments.

## CONCLUSIONS

We describe a novel murine model that reproduces the development of atherosclerosis in the context of HIV infection. Using this model, we demonstrate that Nef is responsible for increased atherosclerosis in HIV-infected animals. This study reinforces Nef's critical role in atherogenesis during viral infection, highlighting it as a potential therapeutic target. Additionally, this model offers a valuable platform to test atherosclerosis treatments in the context of ART-suppressed HIV.

## Supplementary Material

jiaf192_Supplementary_Data

## Supplementary Data

Supplementary materials are available at *The Journal of Infectious Diseases* online (http://jid.oxfordjournals.org/). Supplementary materials consist of data provided by the author that are published to benefit the reader. The posted materials are not copyedited. The contents of all supplementary data are the sole responsibility of the authors. Questions or messages regarding errors should be addressed to the author.

## Notes

***Acknowledgments.*** We thank J. Victor Garcia (University of North Carolina at Chapel Hill, Chapel Hill, NC) for donating wild-type and Nef-deficient HIV-1 JR-CSF variants. All in vivo imaging was carried out in the Georgetown-Lombardi Preclinical Imaging Research Laboratory. We are indebted to Drs Zhihong Yang and Julia Kun (Translational Vascular Medicine Branch, National Heart, Lung, and Blood Institute, Bethesda, MD) for invaluable help with aorta analysis. We thank Dr Jessica Schenck (University California San Diego, San Diego, CA) for the help with animal experiments.

***Financial support.*** This work was supported by National Institutes of Health (grant numbers S10 OD025153 and P30 CA051008 to Georgetown-Lombardi Preclinical Imaging Research Laboratory, R01 HL158305 and P30 AI117970 to Michael Bukrinsky).
