# Supplementary material for: Increased Atherosclerosis in HIV-Infected Humanized Mice Is Caused by a Single Viral Protein, Nef
Source: J Infect Dis. 2025 Apr 16;232(1):e116–25. doi: 10.1093/infdis/jiaf192 (PMC12308672; doi:10.1093/infdis/jiaf192)
Supplement: jiaf192_Supplementary_Data [file jiaf192_supplementary_data.pdf]

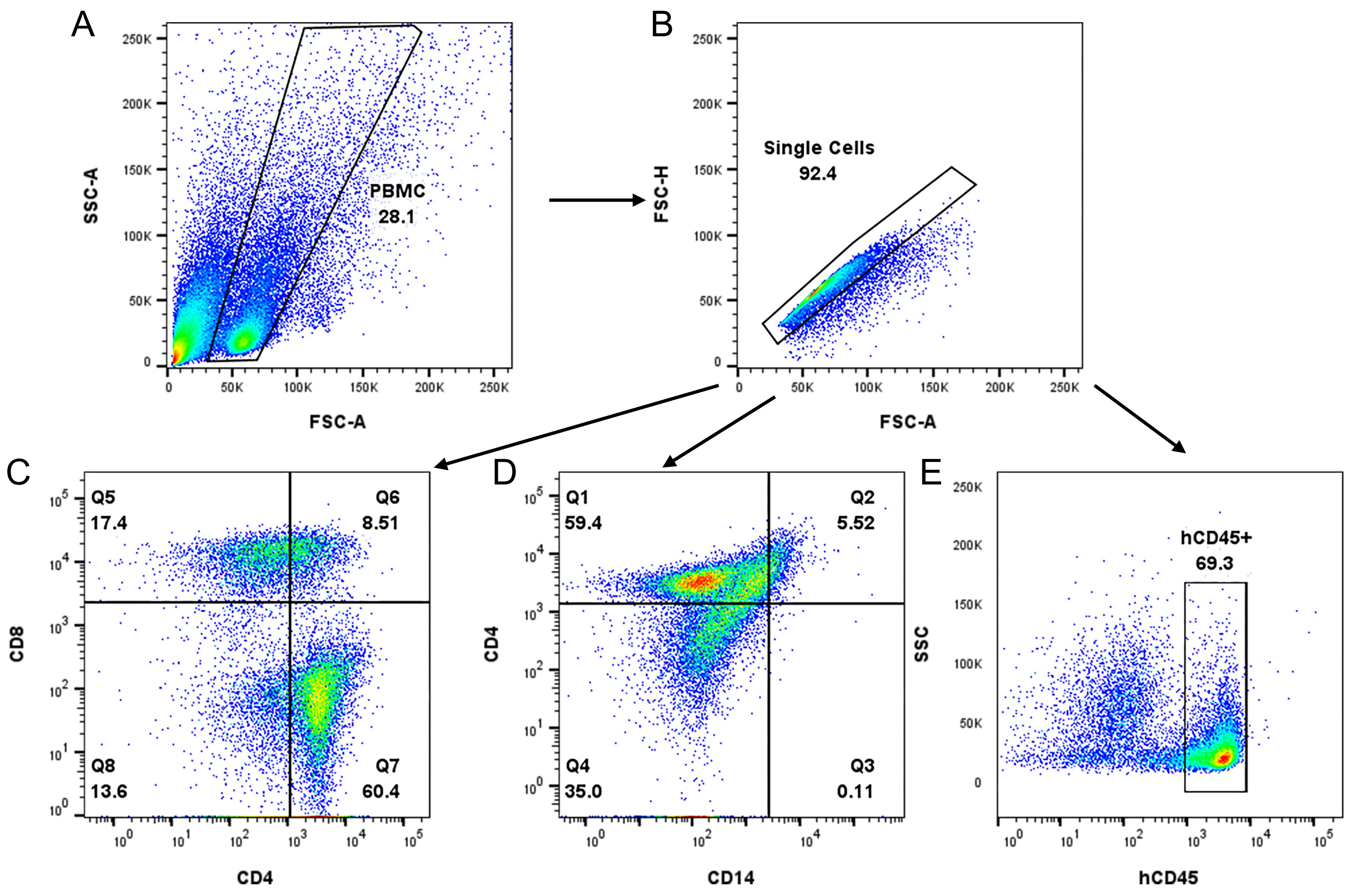

Supplemental Figure S1. Analysis of mouse humanization by flow cytometry. PBMCs isolated from peripheral blood of humanized mice were stained with anti-hCD4, anti-hCD8, anti-hCD14, and anti-hCD45. **A** – gating on PBMCs; **B** – gating on single cells; **C** – hCD8 and hCD4 staining; **D** – hCD4 and hCD14 staining; **E** – hCD45 staining.

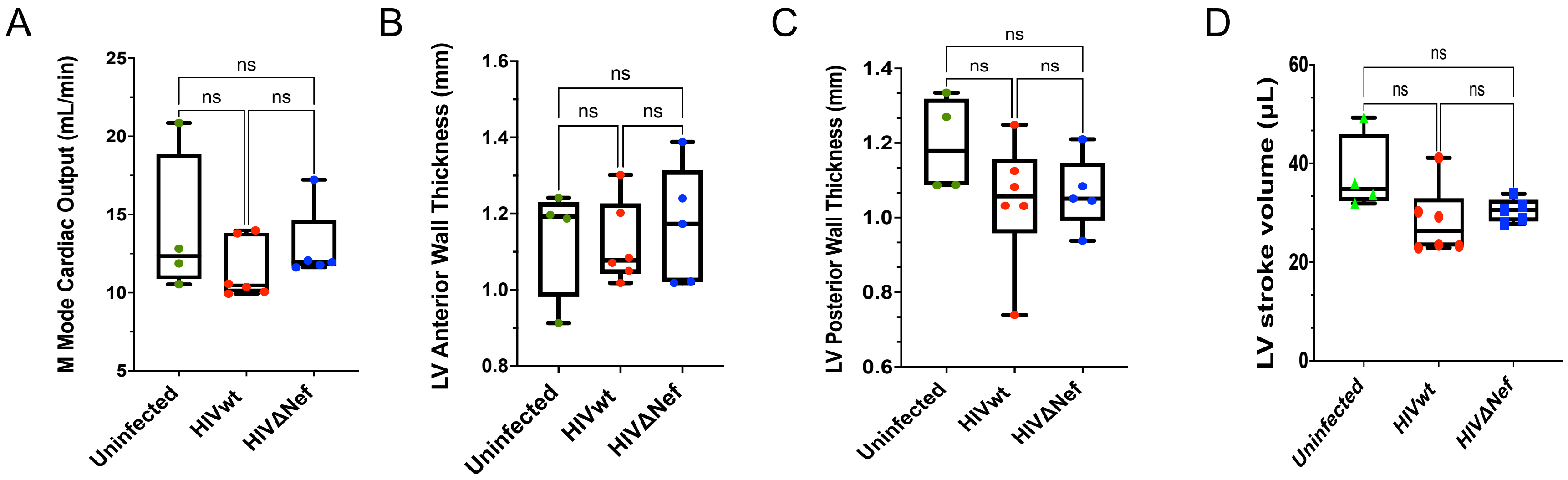

Supplemental Figure S2. Functional analysis by echocardiography. The left ventricle (LV) was measured transversely using M Mode at the midline of the heart proximal to the anterior and posterior papillary muscles. A trace of the endo and epicardial border was made on both the anterior and posterior walls over three successive cardiac cycles and analyzed using the commercially available software Vevo LAB ver. 5.8.2. Data are shown as average +/- SEM. **A** - Cardiac Output calculation (CO = Stroke Volume x Heart Rate). **B** - The average systolic LV anterior wall thickness calculation. **C** - The average systolic LV posterior wall thickness calculation. **D** - The average LV Stroke Volume (SV) calculation (SV = End Diastolic Volume - End Systolic Volume).

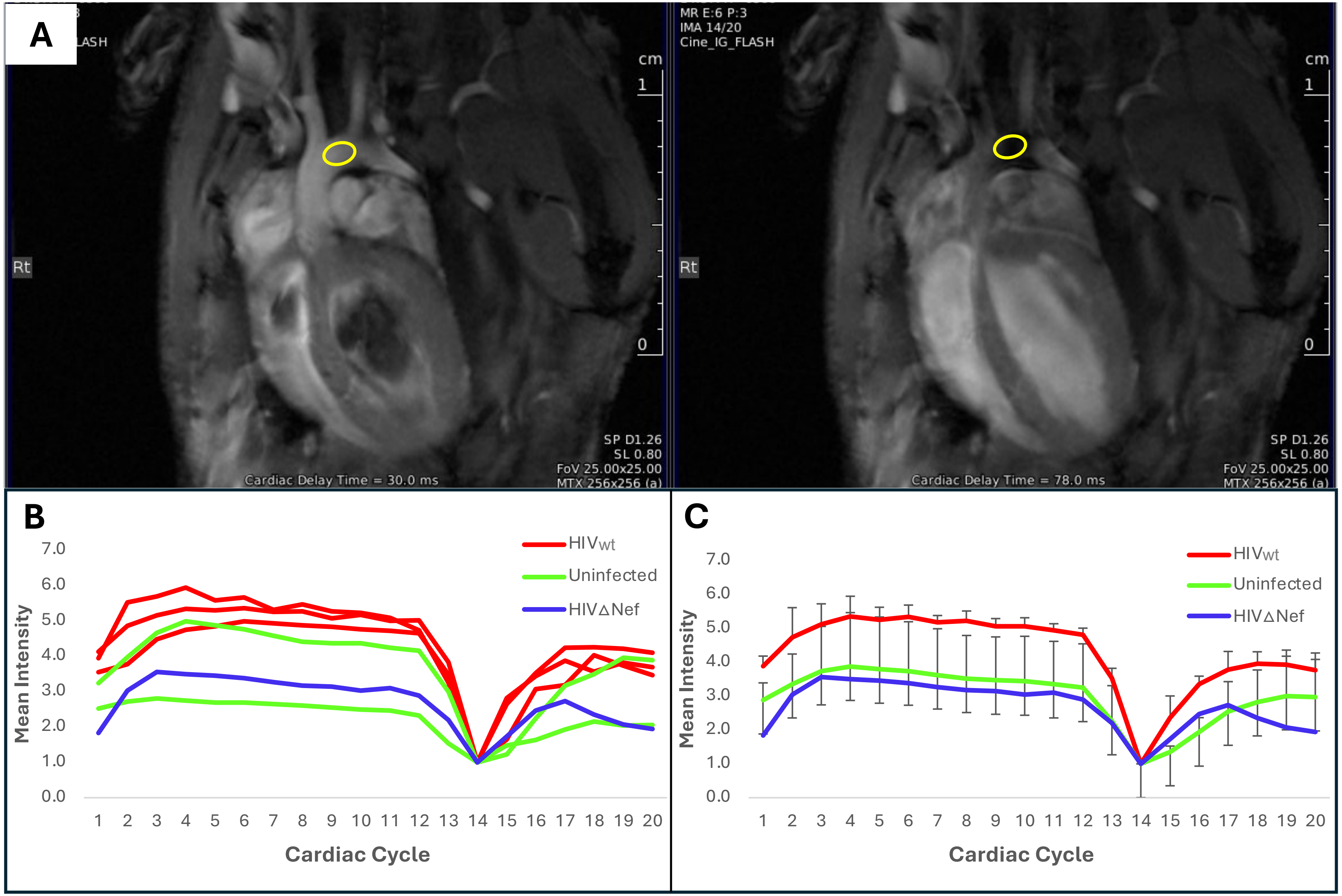

Supplemental Figure S3. Magnetic resonance imaging of the aortic blood flow. A CINE IG FLASH MRI sequence was run for coronal/sagittal visualization of the aortic arch. **A -** In order to assess aortic arch blood flow, the mean intensity of a region of interest (ROI) localized on the aortic arch (yellow ellipse) was measured repeatedly for over a cardiac cycle. The heart and aorta are shown in systole (left panel) and in diastole (right panel). **B -** The aortic arch mean intensities of individual mice are plotted. **C -** The corresponding averaged mean intensities show that the blood flow of the HIV-infected mice was increased compared to the uninfected control mice and mice infected with HIVΔNef, possibly secondary to a narrowed and less elastic atherosclerotic aorta. Error bars represent standard deviation.

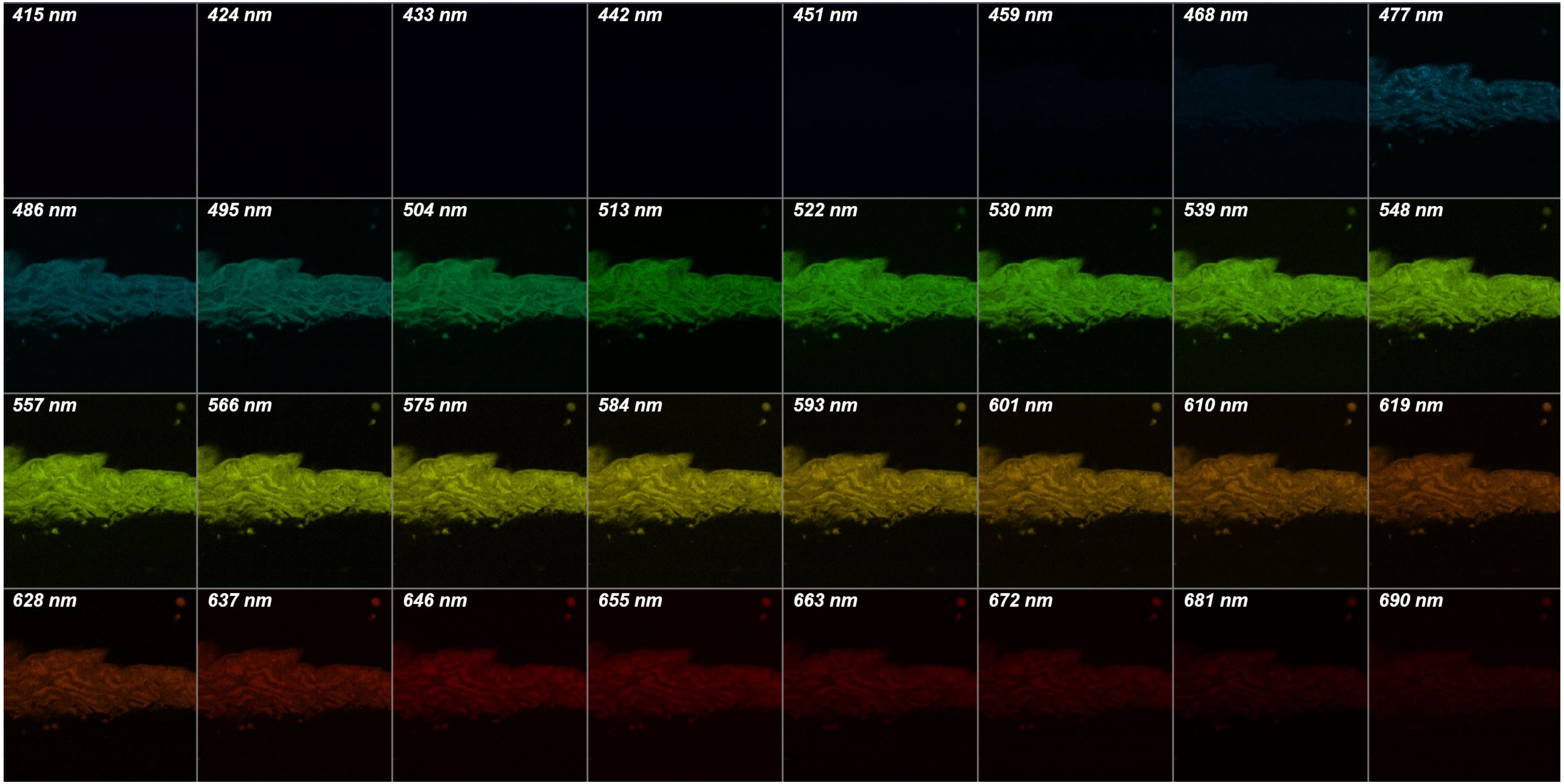

Supplemental Figure S4A. Images of aorta captured with Zeiss/Hamamatsu 32-channel spectral detector and Zeiss 63x/1.4 NA lens. Panels represent the individual unique spectral images obtained in the range between 411-695 nm, upon excitation with a single 960 nm light. The composite image is in Figure S4Ba.

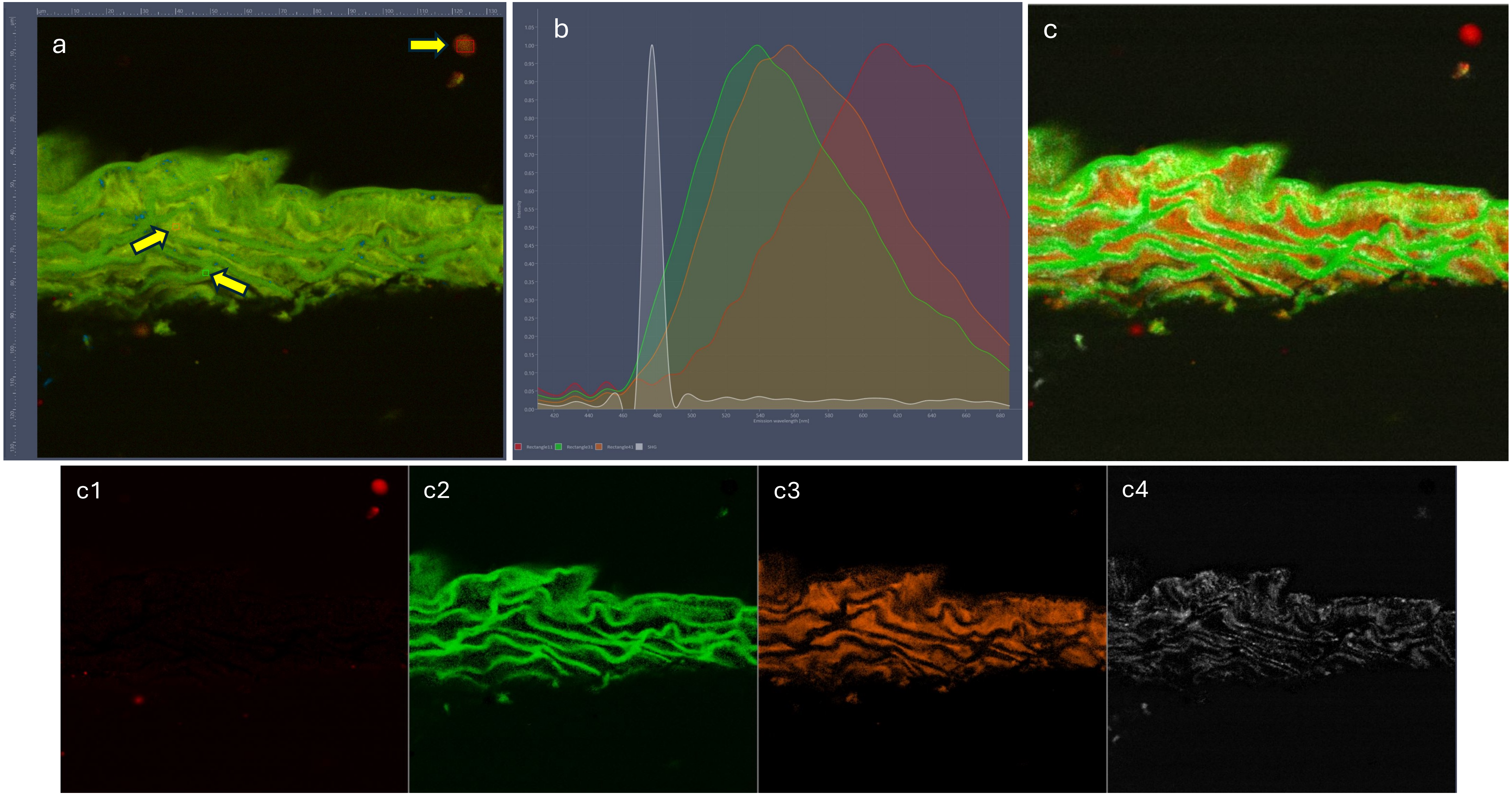

Supplemental Figure S4B. Spectral imaging and linear spectral unmixing workflow. **a, b -** a composite lambda stack of 32 spectrally unique images obtained as in Fig. S4A. Arrows in panel **a** show the location of the spots where the spectral curves for two classes of autofluorescence – green (green curve on **b,** elastic fibers), brown (brown curve on **b**, cellular elements) - and Oil Red O fluorescence (red curve on **b**) were obtained. The SHG curve (grey on **b**), which did not follow Boltzmann distribution of probabilities, was obtained from a different section of the same volume. Please note the slight difference in the composite lambda stack qualitatively discriminating elastin and cellular autofluorescence. **c** - linear spectral unmixing product using the curves in **b** and the image data in **a**. **c1-c4** - single channel images composing the image in **c**. **c1** – Oil Red O, **c2** – elastin autofluorescence, **c3** – cellular components, **c4** – collagen SHG.

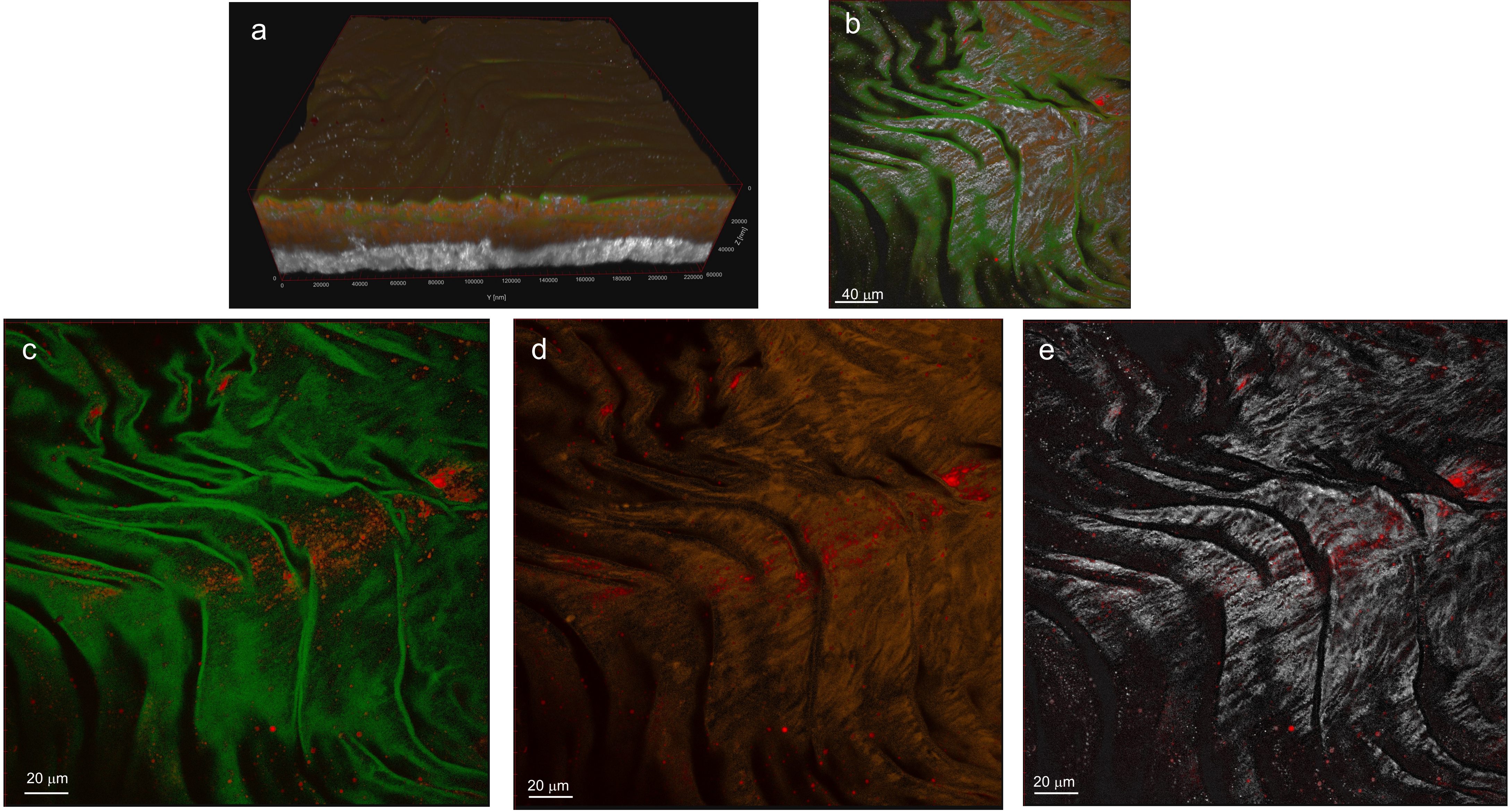

Supplemental Figure S5A. High magnification view of the plaque area in aorta of HIVwt-infected mouse. Representative volume rendering acquired with online linear spectral unmixing using the curves in Fig. S1B. **a** - composite 3D rendering of 4 channels representing autofluorescence deriving from elastic fibers (green) and cellular elements (brown), collagen (SHG, gray), and Oil Red O fluorescence. **b** - a single optical section from the volume in **a**, emphasizing the arrangement of the collagen fibers (gray) relative to the endothelial surface (green). Oil Red O (red) is identified clearly in the layers of the aorta bellow the endothelium. **c** - elastin autofluorescence with Oil Red O fluorescence. **d** - cellular autofluorescence with Oil Red O fluorescence. **e** - collagen SHG with Oil Red O fluorescence. Scale bar, 40 µm in B, 20 µm in C-E.

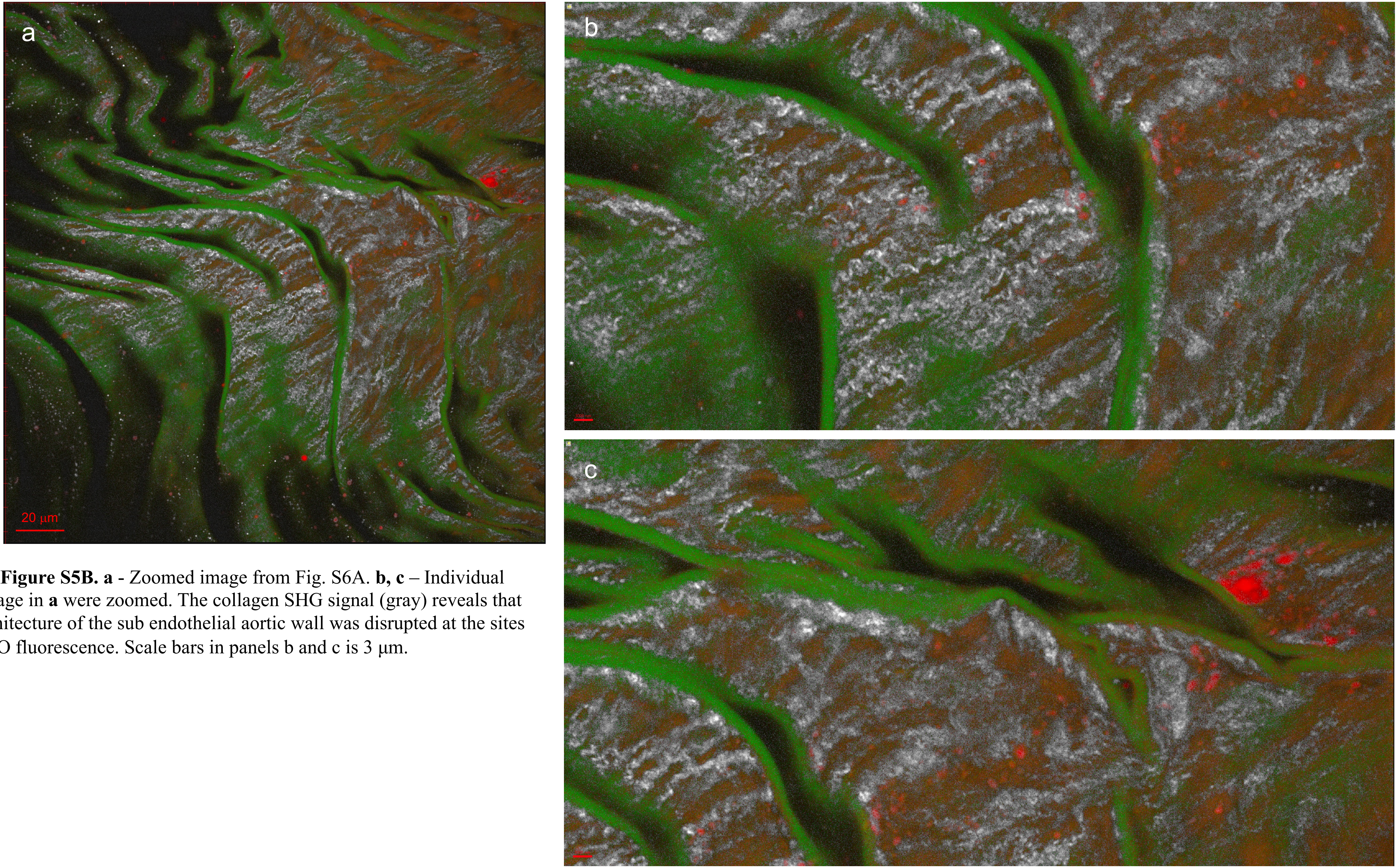

Supplemental Figure S5B. a - Zoomed image from Fig. S6A. **b, c** – Individual areas of the image in **a** were zoomed. The collagen SHG signal (gray) reveals that the normal architecture of the sub endothelial aortic wall was disrupted at the sites of the Oil Red O fluorescence. Scale bars in panels b and c is 3 µm.

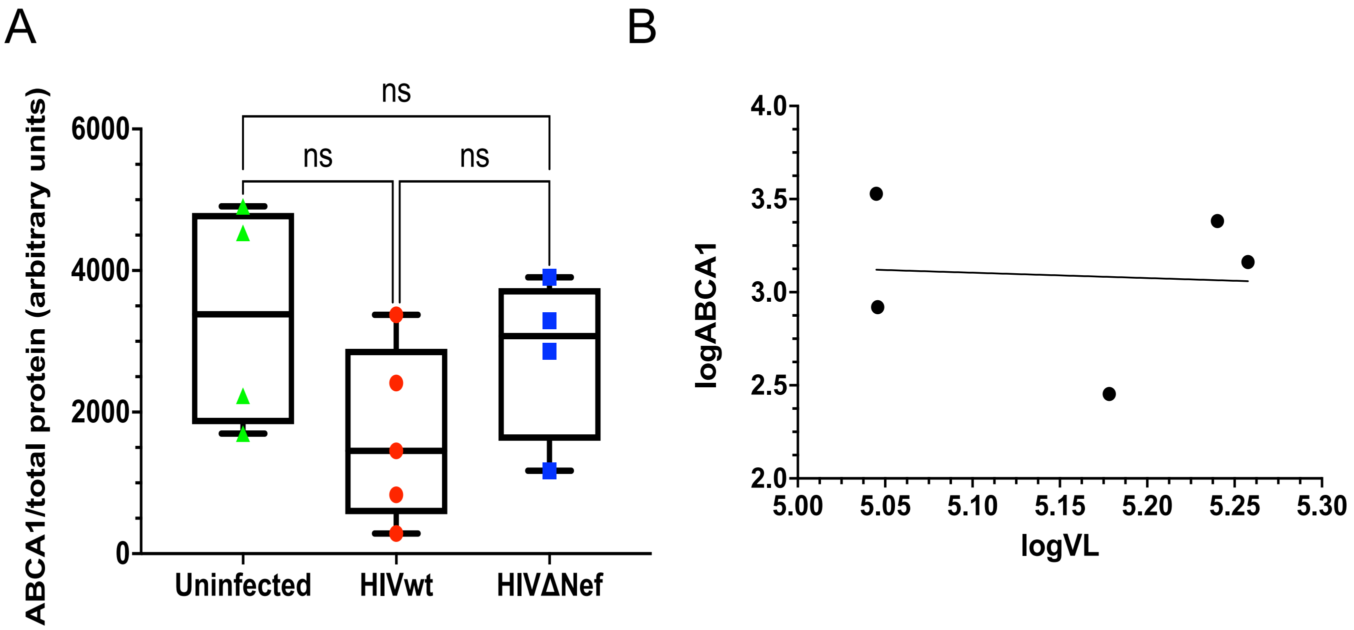

Supplemental Figure S6. Analysis of ABCA1 in the liver. Livers from experimental mice were homogenized and analyzed by Western blot on BioTechne automated Western blot instrument. Results were adjusted to total protein loaded to corresponding lane and are presented as box plots showing mean and SEM **(A).** P values were calculated by ordinary one-way ANOVA with Sidak correction for multiple comparisons. **B** – Linear regression analysis of ABCA1 and VL.

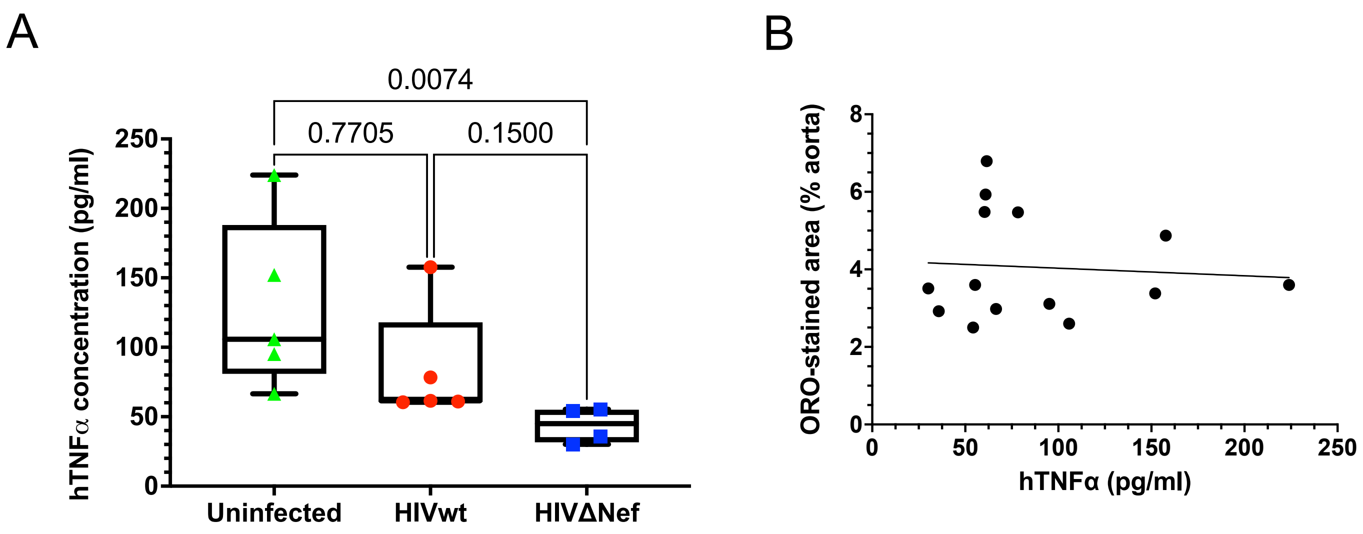

Supplemental Figure S7. Analysis of human TNFα. A - hTNFα was assayed by ELISA in plasma of humanized mice infected with HIV (wt or ΔNef) or uninfected. Results were analyzed by Kruskal-Wallis non-parametric test with Dunn's post-hoc multiple comparisons. **B** – Linear regression analysis of correlation between ORO-stained area and human TNFα concentration.
